# Le tétanos, une maladie infectieuse encore d'actualité à Lomé (Togo)

**DOI:** 10.48327/mtsi.v3i3.2023.273

**Published:** 2023-09-24

**Authors:** Lidaw Déassoua BAWE, Awèréou KOTOSSO, Akouda Akessiwe PATASSI, Bawoubadi ABALTOU, Olivier Pabamé NAORA, Abdou-Razak MOUKAÏLA, Ohouzangbe DOULÉ, Majesté Ihou WATÉBA

**Affiliations:** 1 Service des maladies infectieuses et tropicales, CHU Sylvanus Olympio, Lomé, Togo; 2 Centre hospitalier des armées de Lomé, Togo; 3 École du service de santé des armées de Lomé, Togo

**Keywords:** Tétanos, Vaccination, Lomé, Togo, Afrique subsaharienne, Tetanus, Vaccination, Lome, Togo, Sub-Saharan Africa

## Abstract

**Introduction:**

Le tétanos demeure un problème de santé publique au Togo malgré la mise en place du Programme élargi de vaccination.

**Patients et méthode:**

Il s'agit d'une étude de cohorte rétrospective et descriptive qui s'est déroulée du 1er janvier 2008 au 31 décembre 2018 dans le service des maladies infectieuses et tropicales du Centre hospitalier universitaire Sylvanus Olympio de Lomé sur des cas de tétanos.

**Résultats:**

En 11 ans, 208 cas de tétanos ont été inclus. La fréquence hospitalière dans le service des maladies infectieuses et tropicales du CHU Sylvanus Olympio était de 6,5%. L'âge médian des patients était de 23 ans [13-38 ans] avec une prédominance masculine (81,2%). Les patients étaient des ouvriers (63,5%) et provenaient majoritairement du milieu urbain (65,9%). La vaccination antitétanique n'était à jour que chez 9,3% d'entre eux. Les portes d'entrée étaient dominées par les plaies cutanées (66,8%). La forme généralisée représentait 181 cas. Le sérum antitétanique a été administré chez 191 patients (91,8%), le plus souvent par voie intrathécale (189 patients). Les complications étaient marquées par la surinfection de la plaie (n = 8), un état de choc septique (n = 3), une insuffisance respiratoire aiguë et une nécrose cutanée dans 1 cas respectivement. La létalité était de 27,4%.

**Conclusion:**

La morbidité du tétanos en particulier juvénile reste toujours élevée avec une létalité importante. Il importe donc de mettre un accent particulier sur le volet des rappels vaccinaux.

## Abstract (en)

**Introduction:**

Tetanus still remains a public health problem in Togo despite the existence of the Expanded Program on Immunization.

**Patients and method:**

A retrospective and descriptive cohort study was performed from January 1, 2008 to December 31, 2018 in the infectious and tropical diseases department of Sylvanus Olympio teaching hospital of Lome (Togo) on tetanus cases.

**Results:**

We included 208 tetanus cases accounting for 6.5% of the whole hospitalizations in the infectious and tropical diseases department at this hospital. The median age of the patients was 23 [13-38 years] with male predominance (81.2%). The patients were workers (63.5%) and came mainly from urban areas (65.9%). Tetanus vaccination was only up to date in 9.3% of patients. Gateways were dominated by skin wounds (66.8%). Antitetanus serum was administered in 191 patients (91.8%) mainly through intrathecal route (189 patients (91.1%)). Complications were marked by superinfection of the wound (n=8), septic shock (n=3), acute respiratory failure and skin necrosis in 1 case respectively. The lethality was 27.4%.

**Conclusion:**

The morbidity of tetanus, in particular juvenile morbidity, remains high with significant lethality. It is therefore important to place particular emphasis on the vaccine booster component.

## Introduction

Le tétanos, causé par *Clostridium tetani,* est une maladie infectieuse cosmopolite, de diagnostic exclusivement clinique [2]. Dans les pays industrialisés, le tétanos ne se rencontre que très rarement, le plus souvent chez les personnes âgées. En France, la prévalence demeure faible, 0,28 à 0,5 cas par million d'habitants entre 2002 et 2004 [1]. Dans les pays du sud, en revanche, tous les âges peuvent être touchés, surtout la tranche d'âge de 6 à 30 ans [9], avec un taux de létalité encore élevé de l'ordre de 60% surtout imputable au manque d'équipement des services de réanimation [8].

Le tétanos y demeure un problème de santé publique malgré l'existence du Programme élargi de vaccination grâce auquel l'Organisation mondiale de la Santé (OMS) avait prévu l'élimination du tétanos néonatal et maternel pour 2005 [15].

Au Togo, le tétanos sévit sur un mode endémique et l'on constate une augmentation croissante de la prévalence avec une létalité hospitalière souvent supérieure à 50% [6]. Il représente environ 5% des motifs d'hospitalisation dans le service des maladies infectieuses et tropicales du Centre hospitalier universitaire Sylvanus Olympio de Lomé, après l'infection à VIH et ses maladies opportunistes [13].

Depuis 30 ans, trois études ont été menées sur le tétanos au Togo. La première a porté sur le tétanos néonatal [6], la deuxième concernait l'intérêt de la thérapie intrathécale à base de sérum antitétanique dans la prise en charge du tétanos [14] et la dernière s'est penchée sur la relation entre infection à VIH et tétanos [13]. Bien des années après ces études, nous avons trouvé indispensable de faire un état des lieux du tétanos dans le service des maladies infectieuses et tropicales du Centre hospitalier universitaire Sylvanus Olympio de Lomé où cette affection reste encore fréquente en hospitalisation.

L'objectif principal assigné à ce travail était d'analyser les causes du maintien du tétanos et de faire des recommandations pratiques en vue de son contrôle.

## Matériel et méthodes

Il s'est agi d'une étude rétrospective et descriptive allant du 1^er^ janvier 2008 au 31 décembre 2018 dans le cadre du service des maladies infectieuses et tropicales du Centre hospitalier universitaire Sylvanus Olympio (CHU SO). Service de référence en matière de prise en charge des pathologies infectieuses. Le CHU SO est la plus grande formation sanitaire du Togo avec une capacité théorique d'accueil de 1 264 lits [3].

Nous avons inclus les patients des deux sexes hospitalisés pour tétanos dans le service des maladies infectieuses et tropicales durant la période d'étude. Les patients qui se trouvaient dans le registre d'hospitalisation du service mais dont les dossiers étaient incomplets ont été exclus.

Le diagnostic du tétanos étant clinique, il a été posé devant les signes tels que le trismus, les contractures musculaires généralisées et les paroxysmes.

Le recueil des données a été fait à l'aide d'une fiche préétablie. Les données sociodémogra-phiques (âge, sexe, milieu de résidence, profession), cliniques (antécédents médicaux et chirurgicaux, antécédents vaccinaux existants, porte d'entrée, température, pouls, durée d'incubation et d'invasion, trismus, dysphagie, contractures et paroxysmes), thérapeutiques (isolement sensoriel, antibiotiques, sédatifs, traitement de la porte d'entrée, sérothérapie et vaccination antitétanique) et évolutives (guérison, sortie sans avis médical, décès, séquelles) ont été recueillies à partir des dossiers médicaux des patients et des registres d'hospitalisation.

Tout patient provenant d'un quartier de la capitale ou d'une ville des préfectures du Togo était considéré comme provenant du milieu urbain. Tout patient provenant d'un village ou canton était considéré comme résidant dans le milieu rural.

Les professions identifiées étaient les ouvriers exerçant des activités ou tâches physiques dans un secteur formel ou informel, les commerçants exerçant une activité lucrative et génératrice de revenus, les fonctionnaires ou salariés de la fonction publique, les sans profession regroupant les chômeurs et les retraités, et les étudiants inscrits à l'université ou dans une école supérieure.

Le score pronostique de Dakar a été utilisé pour déterminer le score des patients à leur admission pour tétanos.

Le statut vaccinal des patients a été déterminé à l'interrogatoire et/ou sur la présentation du carnet de vaccination.

Le sérum antitétanique était administré dès que le diagnostic du tétanos a été posé à la dose de 1500 unités internationales. La voie d'administration était fonction du score pronostique de Dakar. La voie intrathécale était utilisée à partir d'un score > 2.

## Résultats

### Aspects épidémiologiques

#### Fréquence

Un total de 3 464 admissions toutes pathologies confondues a été enregistré dans le service des maladies infectieuses et tropicales du CHU SO durant la période d'étude. On dénombrait 225 cas de tétanos hospitalisés, soit une fréquence hospitalière de 6,5%. La moyenne annuelle d'admission des cas de tétanos était de 20 cas. Les dossiers qui répondaient aux critères d'inclusion et exploitables étaient au nombre de 208.

#### Répartition des patients selon les années

**Figure 1:**
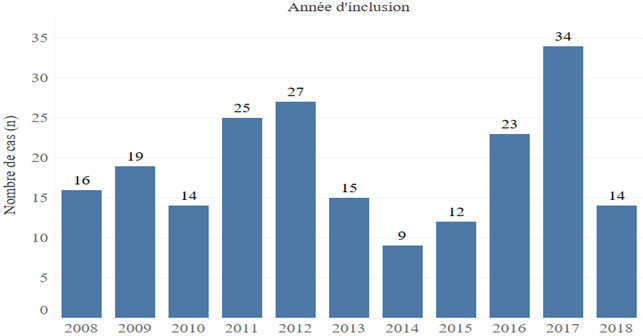
Répartition des cas de tétanos selon les années dans le service des maladies infectieuses du CHU Sylvanus Olympio Distibution of tetanus cases by year in the infectious and tropical diseases department of Sylvanus Olympio teaching hospital

La Figure 1 montre que l'année 2017 a enregistré le plus grand nombre de cas de tétanos avec 34 patients hospitalisés.

#### Répartition des patients selon l'âge et le sexe

L'âge médian [IQ] était de 23 [13-38] ans, avec une prédominance masculine (n = 169), soit un sex-ratio H/F de 4,3. On notait 4 cas de tétanos néonatal (1,9%).

#### Répartition des patients selon la provenance et la profession

La majorité des cas de tétanos hospitalisés (69,5%) provenaient du milieu urbain (n = 137); les ouvriers étaient les plus exposés au risque de tétanos soit 63,5% (n = 132), suivis par les élèves/étudiants (n = 37), les sans profession (n = 34), les fonctionnaires (n = 3) et les commerçants (n = 2).

#### Profil vaccinal des patients

Parmi les 208 patients de notre série, excepté les 4 nouveau-nés, seuls 19 patients étaient correctement vaccinés contre le tétanos avant la blessure.

#### Porte d'entrée

Les plaies cutanées retrouvées chez 139 patients (66,8%) étaient les portes d'entrée majoritaires. La porte d'entrée n'a pas été retrouvée dans 14,9% des cas (Tableau I). Les circonstances des blessures étaient marquées par des traumatismes (accidents de la voie publique, accidents domestiques, accidents au cours de jeux), des piqûres d'objets, des brûlures, des morsures animales, des scarifications et des portes d'entrée iatrogènes suite à des soins dans une formation sanitaire (suture, injection intramusculaire).

**Tableau I:** Répartition des patients selon la porte d'entrée du bacille tétanique Distribution of patients according to the portal of entry of the bacillus

Porte d'entrée	Effectif (n)	Pourcentage (%)
**Plaie cutanée**	139	66,8
**Non retrouvée**	31	15,0
**Chirurgicale**	22	10,5
**Injection intramusculaire**	9	4,3
Autres*	7	3,4
**Total**	208	100

* otogène (n = 4); ophtalmique (n = 1); anale (n = 1); scarifications (n = 1)

#### Siège de la porte d'entrée ou plaie

Les plaies siégeaient aux membres pelviens dans 101 cas soit 48,6% (Tableau II).

**Tableau II:** Répartition des cas de tétanos selon le siège de la plaie dans le service des maladies infectieuses et tropicales du CHU Sylvanus Olympio Distribution of tetanus cases according to the site of the wound in the infectious and tropical diseases department of Sylvanus Olympio teaching hospital

	Effectif (n)	Pourcentage (%)
**Membre pelvien**	101	48,6
**Tête et cou**	28	13,5
**Membre thoracique**	25	12,0
**Pelvis**	11	5,3
**Abdomen**	9	4,3
**Thorax**	3	1,4
**Non précisé**	31	14,9
**Total**	208	100

### Aspects cliniques

#### Délai de consultation

Le délai de consultation après le début des signes n'a pas été précisé chez 50 patients. Pour le reste des patients, le délai médian était de 3 jours.

La médiane de la période d'incubation était de 8 jours. La durée d'incubation n'a pas été précisée chez 75 patients.

La durée de la période d'invasion n'était pas précisée chez 45 patients. Elle était inférieure à 48 heures chez 40,2% des patients. La durée d'invasion moyenne était de 63 heures avec un écart type de 66 et des extrêmes de 24 et 204 heures.

#### Signes cliniques

Le trismus (n = 173), la raideur de la nuque (n = 181) et les paroxysmes (n = 104) étaient les signes cliniques les plus fréquents (Tableau III).

**Tableau III:** Répartition des cas de tétanos selon les signes cliniques dans le service des maladies infectieuses et tropicales du CHU Sylvanus Olympio Distribution of tetanus cases according to clinical signs in the infectious and tropical diseases department of Sylvanus Olympio teaching hospital

**Signes cliniques**	**Effectif (n)**	**Pourcentage (%)**
**Trismus**	173	83,2
**Signe de l'abaisse-langue captif d'Armangaud**	35	16,8
**Dysphagie**	34	16,3
**Raideur de la nuque**	181	87,0
**Contractures musculaires généralisées**	181	87,0
**Opisthotonos**	181	87,0
**Paroxysmes**	104	50,0

### Aspects thérapeutiques

#### Traitement symptomatique

Tous les patients ont reçu un sédatif; le diazépam chez 196 patients et le phénobarbital administré à 16 patients.

Les mesures de réanimation ont été nécessaires chez 21 patients; 19 patients ont nécessité une ventilation au masque et 2 trachéotomies ont été pratiquées.

#### Traitement spécifique

La désinfection de la porte d'entrée a été réalisée chez 102 patients.

Le sérum antitétanique associé à un corticoïde a été administré à 191 patients soit 91,8%, principalement en intrathécale chez 174 patients (91,1%).

Tous les patients ont reçu une antibiothérapie. La pénicilline G était utilisée dans la majorité des cas (92,3%), seule ou associée au métro-nidazole dans 88% des cas.

À titre préventif, le vaccin antitétanique a été administré chez 80,3% des patients.

### Aspects évolutifs

La durée médiane d'hospitalisation était de 8 [3-14] jours.

L'issue a été favorable chez 128 patients (61,5%) dont le score pronostique médian de Dakar était de 1 [1-2] point. Huit patients sont sortis contre avis médical (3,8%) et 4 patients se sont évadés (1,9%). Le mode de sortie n'a pas été précisé chez 11 patients (5,3%). La létalité était de 57 décès soit 27,4%, dont le score pronostique médian de Dakar était de 2 [1-3] points.

Des complications ont été enregistrées chez 15 patients : surinfection de la porte d'entrée (8 cas), arrêt cardiovasculaire (4 cas), choc septique (3 cas), insuffisance respiratoire aiguë (1 cas) et nécrose cutanée (1 cas).

Après analyse, il ressort que le mode évolutif du tétanos était fonction du statut vaccinal avant la survenue du tétanos, de la prévention antitétanique après la blessure et de l'administration de sérum antitétanique associé aux corticoïdes (Tableau IV).

**Tableau IV:** Facteurs associés à l'évolution du tétanos Factors associated with the evolution of tetanus

Variables	Guérison	Décès	P value
**Statut vaccinal non à jour avant la survenue du tétanos**			
oui	120 (93,75%)	46 (80,7%)	0,002
non	8 (6,25%)	11 (19,3%)	
total	128 (100%)	57 (100%)	
**Circonstance de survenue de la porte d'entrée**			
traumatisme	120 (93,75%)	48 (84,2%)	0,4
non traumatique	8 (6,25%)	9 (15,8%)	
total	128 (100%)	57 (100%)	
**Prévention antitétanique après la blessure**			
oui	11 (8,6%)	1 (1,8%)	0,03
non	117 (91,4%)	56 (98,2%)	
total	128 (100%)	57 (100%)	
**Administration de sérum antitétanique + corticoïde**			
oui	128 (100%)	44 (77,2%)	0,001
non	0 (0%)	13 (22,8%)	
total	128 (100%)	57 (100%)	
**Voie d'administration du sérum antitétanique + corticoïde**			
intrathécale	122 (95,3%)	42 (95,45%)*	0,4
intramusculaire	6 (4,7%)	2 (4,55%)*	
total	128 (100%)	44(100%)*	

* 44 patients parmi les 57 décès ont reçu le sérum antitétanique + corticoïde

#### Facteurs associés à la létalité

La probabilité de décès était significativement liée à la présence de complications (80%, p < 0,0001). La létalité était plus élevée chez les patients présentant au moins une pathologie associée mais cette différence n'était pas significative (p = 0,185) comme le montre le (Tableau V).

**Tableau V:** Facteurs sociodémographiques et cliniques associés à la létalité Sociodemographic and clinical factors associated with lethality

	Décès						
	Oui (N=57)		Non (N=134)		Non précisé (N=17)		P value
	n	%	n	%	n	%	
**Sexe**							0,635
féminin	13	33,3	23	59,0	3	7,7	
masculin	44	26,0	111	65,7	14	8,3	
Âge							0,470
≤ 23	28	25,7	74	67,9	7	6,4	
> 23	29	29,3	60	60,6	10	10,1	
**Pathologies associées**							0,185
oui	16	38,1	24	57,1	2	4,8	
non	41	24,7	110	66,3	15	9,0	
**Complications**							< 0,0001
oui	12	80,0	2	13,3	1	6,7	
non	45	23,3	132	68,4	16	8,3	

## Discussion

Le maintien du tétanos au Togo, en particulier dans la région sanitaire du Grand Lomé et de ses environs, se traduit par une forte prévalence au sein des populations à faible revenu comme les ouvriers, à faible niveau d'étude (primaire et non scolarisé), jeunes, et en absence de prévention antitétanique après une blessure.

Les limites de cette étude sont marquées d'abord par son caractère rétrospectif donc comportant des données recueillies selon des méthodes différentes, non vérifiables, parfois absentes. Néanmoins, nous avons considéré que les données recueillies présentaient une bonne fiabilité.

La prédominance du tétanos au sein de la population jeune et masculine peut s'expliquer par l'importance de la jeunesse de la population dans les pays à faible revenu comme en témoignent les pyramides des âges. Cette population masculine jeune est très active et travaille sans couverture sanitaire ni sécurité sur les lieux de travail, particulièrement dans le secteur informel. De plus, plusieurs cas de tétanos en milieu urbain peuvent être liés à l'environnement, là où l'urbanisation n'est pas contrôlée, exposant les populations aux objets tranchants ou piquants lors des blessures. Les pratiques traditionnelles de prise en charge des plaies en absence de prévention antitétanique sont également des facteurs explicatifs.

De moins en moins de tétanos devraient être observés en milieu urbain du fait de l'existence des services de santé pour la prévention, sous réserve que les populations s'y rendent ! Des erreurs professionnelles ont été notées, comme l'absence de prophylaxie antitétanique dans certaines formations sanitaires où des patients ont consulté.

La sérothérapie antitétanique intrathécale expérimentée dans plusieurs études [5, 14] a montré son efficacité dans le traitement du tétanos, comparée à l'administration en sous-cutanée ou en intramusculaire. Cette efficacité a **été** confirmée dans une étude menée par Watéba *et al.* [14]. Deux groupes de patients répartis de façon aléatoire ont été constitués. Un groupe de 17 patients a reçu 1500 UI de sérum antitétanique par voie intrathécale associé à 1,5 g/j de métronidazole en intraveineuse avec un taux de létalité de 12%. Dans le second groupe de 25 patients traités par une dose de 9000 UI de sérum antitétanique (4500 UI en sous-cutané et 4500 UI en intramusculaire), non combiné systématiquement au métronidazole, la létalité a été de 52%. La durée d'hospitalisation, respectivement 7,4 ± 2,1 jours et 19 ± 6,3 jours, confirmait ces résultats [14].

Le taux de guérison observé est fonction de plusieurs critères comme le score pronostique de Dakar bas, le délai d'administration du sérum antitétanique et l'absence de complications. Dans notre série, une faible proportion n'a pas reçu le sérum antitétanique pour plusieurs raisons dont le manque de moyens financiers pour honorer les ordonnances, ce qui retarde la prise en charge.

En comparant nos résultats avec ceux d'autres études régionales, nous constatons également que l'âge relativement jeune des patients victimes de tétanos, généralement inférieur ou égal à 30 ans, a été rapporté dans deux études rétrospectives [9, 12].

Bien que le taux de guérison soit à 50% dans plusieurs études [7, 11, 14], la létalité du tétanos reste élevée malgré la sérothérapie intrathécale et la prise en charge en unité de soins intensifs [10, 14, 16].

La durée moyenne d'hospitalisation dépasse deux semaines [4, 12]. Elle est proportionnelle au score des patients à l'admission.

La prise en charge en hospitalisation étant entièrement aux frais du patient, elle représente un fardeau pour les familles surtout quand l'hospitalisation se prolonge. Cette situation pousse les patients et les accompagnants à quitter l'hôpital sans prévenir ou contre avis médical.

## Conclusion

Le tétanos est une maladie encore fréquente malgré l'existence d'un vaccin efficace. Au Togo, particulièrement dans la région sanitaire du Grand Lomé, il demeure un problème de santé publique du fait de sa fréquence, de sa gravité et de sa létalité au sein de la population masculine et jeune. Les malades n'ont pas toujours recours à la vaccination en cas d'exposition. À l'instar du tétanos néonatal et maternel que l'OMS s'était engagée à éliminer, le tétanos devrait être inscrit dans la liste des maladies régies par le système de surveillance intégrée de la maladie et de la riposte. Un accent devrait également être mis sur l'information, l'éducation et la communication à l'endroit de la population pour une meilleure adhérence au calendrier vaccinal.

## Contribution Des Auteurs

BAWE Lidaw Déassoua : co-directeur du travail et rédacteur de l'article. KOTOSSO Awèréou : relecture de l'article et contribution dans l'ensemble. BALTOU Bawoubadi : relecture de l'article et contribution dans l'ensemble. PATASSI Akouda Akessiwe : relecture de l'article et contribution au niveau de la méthodologie. MOUKAÏLA Abdou-Razak : analyses statistiques. NAORA Olivier Pabamé : initiateur et concepteur du travail. DOULE Ohouzangbé : identification des dossiers aux archives pour leur exploitation. WATEBA Majesté Ihou :a dirigé le travail

## Liens D'intérêts

Les auteurs ne déclarent aucun conflit d'intérêts.
